# Penetrating thorn in the heart complicated by infective endocarditis

**DOI:** 10.1002/ccr3.5266

**Published:** 2022-01-11

**Authors:** Salah Eldin M. E. Hassan, Mohamed Alamin Ahmed, Sabir Taha Hussein, Abdallah Elamin Elsheikh, Moh. Mah. Fadel Alla Eljack, Mazin Salah‐Eldien Hassan Haroun, Khabab Abbasher Hussien Mohamed Ahmed, Mohammed Eltahier Abdalla Omer, Ghassan Elfatih Mustafa

**Affiliations:** ^1^ Department of Cardiothoracic Surgery Medani Heart Diseases and Surgery Center ElGezira Sudan; ^2^ Department of Pediatric Cardiology Medani Heart Diseases and Surgery Center ElGezira Sudan; ^3^ University of Bakht Alruda Medani Heart Diseases and Surgery Center ElGezira Sudan; ^4^ Faculty of Medicine University of Khartoum Khartoum Sudan; ^5^ Medicine Department Gadarif University Faculty of Medicine and Health Sciences Al Qadarif Sudan

**Keywords:** cardiopulmonary bypass, echocardiography, foreign body, infective endocarditis, thorn

## Abstract

A 3‐year‐old child presented with recurrent chest pain for 3 months, echocardiography showed a thorn inside the left ventricle, the patient was diagnosed with foreign body complicated with infective endocarditis and received proper treatment, and operation was performed after inflammatory reaction subsided.

## Graphical abstract

A Penetrating through complicated with Infective Endocarditis in a 3‐years old child.

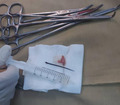

## 1 INTRODUCTION

A foreign body can enter the heart either directly through a penetrating trauma or through migration of a distal fragment from peripheral vessels. However, all of these events are considered to be of very rare occurrence with possible complications to occur including infective endocarditis, cardiac tamponade, and pneumothorax.^1^, ^2^ Infective endocarditis (IE) is a cardiac infection that affects the endothelium. Its annual incidence is 3–10 per 100,000 people, with a fatality rate of up to 30% after 30 days. Because of the variety of causative microorganisms, underlying cardiac diseases, and pre‐existing comorbidities, the clinical presentation of IE is exceedingly diverse. It can appear as an acute, subacute, or chronic condition.^5^

Our case is considered to be the first case documented worldwide of a penetrating thorn in the heart that is complicated with infective endocarditis for which it was first medically treated and then removed through cardiopulmonary bypass surgery.

## 2 CASE REPORT

**FIGURE 1:**
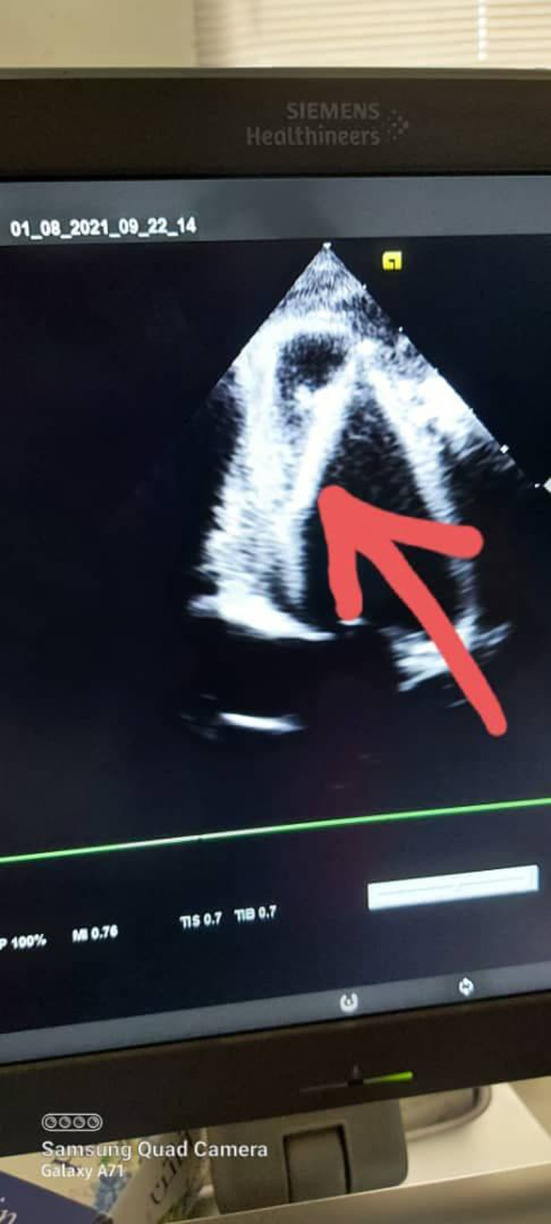
Echocardiography showing a thorn (arrow) within the left ventricle that is penetrating from the apex posteriorly

**FIGURE 2:**
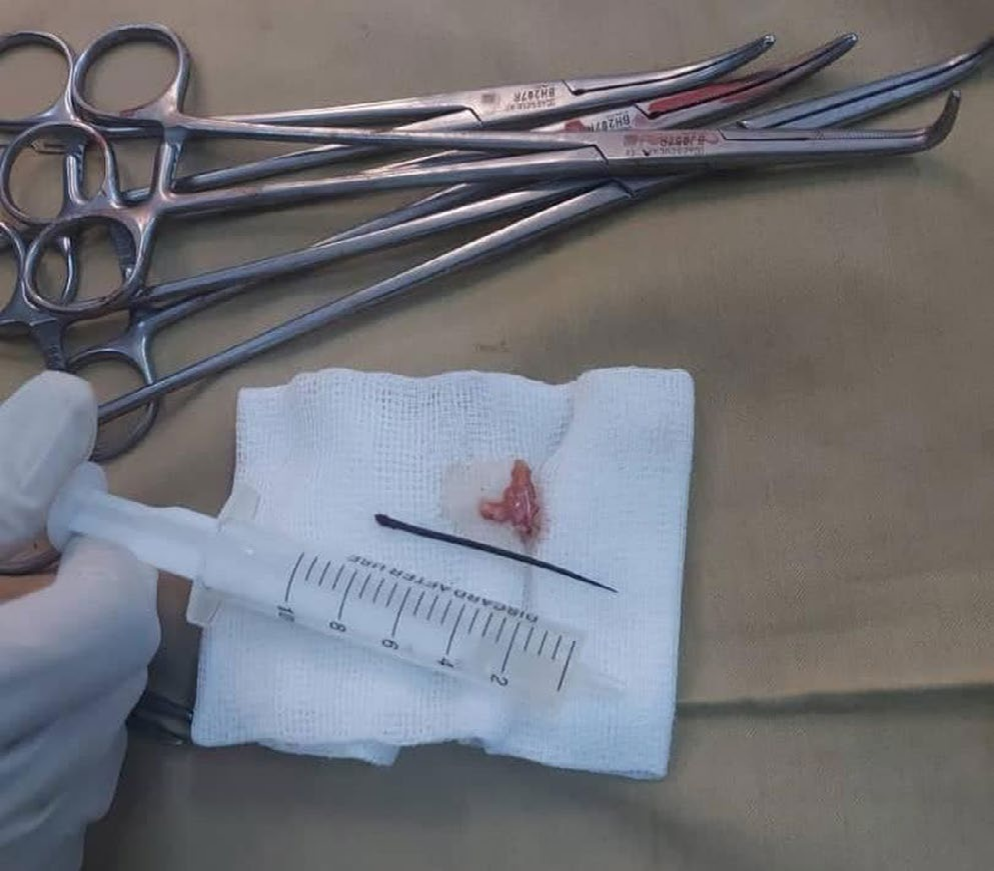
A big slider thorn is shown after being extracted from the patient

A 3‐year‐old child of average body mass index and normal developmental milestones with a history of falling down on his abdomen was referred to the pediatric cardiology department with recurrent chest pain for 3 months that was not managed despite seeking many medical experts. The X‐ray revealed no findings, and he was treated with simple analgesia. Echocardiography showed a thorn within the left ventricle that is penetrating from the apex posteriorly covered by a big clot anteriorly that measures (15 × 11 cm) and was diagnosed as a foreign body that was complicated by infective endocarditis (Figure 1). The patient was put on the medical treatment of infective endocarditis according to the international regimen for 2–3 weeks, followed by inflammatory marker follow‐up, and then, he was shifted to the operation room to undergo median sternotomy, which showed normal pericardium apart from adhesions between chest and diaphragm. The patient was put on bypass after the heart was arrested; adhesions were completely removed; then, the definitive body was revealed (Figure 2). Foreign body reaction and a whole of a big slider thorn emerged. Transatrial approach through the left atrium and then through mitral valve with organized thrombus was adopted, antibiotic solution was applied, mitral valve was checked, and then, we closed. The tricuspid valve was examined and was found to be normal, so the right atrium was closed. We debrided the place of foreign body entry; then, we closed using pericardium strips of two layers that were warmed and separated. Hemostasis was secured, and the chest was closed with two chest tubes. The patient was sent to pediatric ICU under cover of broad‐spectrum antibiotics, antifungals, and analgesia on need. The child was successfully extubated on the next day and was clinically followed up for 1 week, with laboratory markers, and then was sent home after an echo was performed on the day of discharge, which showed a completely normal heart. The patient was told to be seen at refer clinic after 1 month of discharge.

## 3 DISCUSSION

Penetrating cardiac injury in childhood is extremely rare. The objects usually are needles, bullets, grenade acupuncture, or sometimes sewing needles.^2^ In our case, the patient had symptoms of infective endocarditis. To our surprise, there was no cardiac tamponade after trauma as we assume the blood will drain to the left pleural cavity. This is an unusual case in that the thorn directly caused infective endocarditis. The thorn was embedded in the left ventricle, penetrating from the apex posteriorly. As it settled there, it was covered by a big clot anteriorly and adhesions were formed. Management for such cases is very difficult and case dependent. As data concerning foreign bodies in the heart is limited, hence no specific accredited guidelines or recommendations are available. In symptom‐free patients, surgery depends on the type and place of the foreign body. Patients having foreign bodies completely embedded or in the pericardial space or in the myocardium usually remain asymptomatic for a while.^3^ The surgical approach for removing the foreign body is individualized according to the type of foreign body.^4^ Our patient was overall stable, so first we focused on treating infective endocarditis to prevent life‐long complications and to make sure the patient is in the best possible condition for the surgery. Although the risk was high, surgery with bypass was indicated to avoid a life‐threatening situation. Possible complications for such cases vary, and all are life‐threatening if the patient is left untreated. Infective endocarditis and the presence of a foreign body in the heart both contribute to thrombosis and then fatal embolization. Also, the foreign body can cause injury to the surrounding vessels, and heart rhythm can be disturbed.^2^

To our knowledge and recent literature, this is the first case with such a presentation of a thorn with this measurement that is complicated by infective endocarditis.

## 4 CONCLUSIONS

Foreign bodies in the heart are rare but have high mortality and morbidity rates. Proper investigations (such as an echocardiogram), proper treatment for infection, and accurate timing for surgery are keys to patient survival.

## CONFLICT OF INTEREST

All authors declare that there are no conflicts of interest.

## AUTHOR CONTRIBUTIONS

All authors participated in planning the study, data collection, results, and discussion sections.

## ETHICAL APPROVAL

Ethical approval was obtained from the Sudan State Ministry of Health.

## CONSENT

Considering the child was 3 years old, both written and verbal consents were obtained from his guardians (parents) in accordance with the general preoperative guidelines and consent to be the case of interest in this article.

## Data Availability

The data that support the findings of this study are available from the corresponding author upon reasonable request.
